# Our New York Letter

**Published:** 1889-03

**Authors:** 


### Correspondence.

### OUR NEW YORK LETTER.

DR. LECLERCQ—NEW DRUGS AND THEIR USE—THE DULL CURETTE IN PUERPERAL SEPTICAEMIA—THE MIDDLETON GOLDSMITH LECTURE.

*To Editors Atlanta Medical and Surgical Journal:*

At a recent session of the Academy of Medicine, Dr. Schuman LeClercq, of Carlsbad, was present and read a very interesting paper on the influence of Carlsbad water on uric acid excretion. He spoke first of the beneficial use of the waters in gout, and described in detail a series of experiments performed upon himself, which showed that the amount of uric acid excreted daily is upon the whole unaltered by drinking the Carslbad water. He lost in bodily weight, which he considered due to the quality of food and the exercise taken, and perhaps to the high temperature of the water. It is proved, he believed, that the indication afforded by the uric acid secretion is not a reliable index of the amount formed in the body, and the beneficial effect of a remedy in uric acid diathesis cannot possibly be demonstrated by analyzing the urine. Considered clinically, the influence of a course of Carlsbad waters in these cases points in two directions. First, those symptoms supposed to be caused by the "materia peccauns" in the blood or organs, either improve or disappear. Among these are the albuminaria, hypochondria, etc. These troubles, we say, are due to excess of uric acid in the blood and the warm alkaline water favors its discharge. The urine becoming alkaline and diluted removes the surplus of acid in the blood. Secondly, the gouty seizures become less frequent or subside. Here the cause of the disease, he said, must be investigated. One of the functions of the liver being the synthetic formation of uric acid, it is possible that the sodium sulphate contained in Carlsbad waters, which stimulates the secretion of bile, may also affect the formation of uric acid. Dr. LeClercq thought that faulty stomach digestion promoted the gouty diathesis, and hence the dietetic part of the Carlsbad cure was so important. The topical action of the water on the stomach and liver might also account for its beneficial effects. The water being hot tends to increase all the secretions, and excretory matters which might otherwise be retained are eliminated. The bathing and exercise which form a part of the cure, naturally tended to exert a favorable influence in just these parts, the mud bath being, he said, a most agreeable form of poultice. These waters being alkaline and diuretic, possess a prophylactic value, as the gouty attacks, he believes, are caused by a diminished alkalinity of the blood.

Dr. F. N. Otis, in discussing this paper, related his own experience, having undergone a course of treatment at Carlsbad last summer. He had suffered from gout and gouty dyspepsia, accompanied with mental dullness, and was unable to attend properly to his practice. He began treatment by taking seven glasses of the water at intervals during the forenoon. Starchy food was excluded from his dietary, and he drank two glasses of claret a day. He exercised very little, yet lost thirty pounds in a month, and became quite weak. He felt debilitated for a month after leaving Carlsbad, but slowly gained in flesh and strength, and now his condition is better than it has been for a number of years. There was something, he said, in the water which caused the elimination of effete material from the system, and this was just what the cases under consideration required.

During the past few years many new remedies have been added to our already overstocked list, and several novel plans for treating diseases and injuries have been introduced. Some of these innovations have certainly proved of undoubted value, but it cannot be said that many of our old stand-bys have been replaced, at least judging from the present practices in New York.

Antipyrine still holds its own as a remedy for all kinds of headaches, but as an anti-pyretic it is used to a much lesser extent than formerly. Most of our prominent men have discarded it in typhoid fever and it certainly is not taking the place of opium in the relief of pain, or supplanting the salycilates in rheumatism. The hypnotic, sulfanol, is being employed considerably, and with very good results in some cases. True, its use is limited, but as a safe remedy in the majority of cases of sleeplessness from worriment or general nervousness, it appears to be just what is required. Although so much has lately been said against iodoform as an antiseptic, still a great many good surgeons here seem to like it, and in many of the large hospitals the yellow gauze and the powder blower are in daily use. The subiodide of bismuth and a few other substitutes have met with more or less approval, which some of them certainly deserve. Some of the hospitals are now making extensive use of creolin as an antiseptic, in 1 and 2 per cent. solutions, or even stronger. Dr. L. A. Stimson says it seldom irritates the skin and that it is a good antiseptic, and will arrest parenchymætous hæmorrhage and hasten cicatrization. It is also a good deodorizer and is highly recommended for cancerous surfaces which emit a disagreeable odor. At the same time the standard solutions of the bichloride of mercury are used still, almost as much as ever.

Dr. E. H. Grandin of this city, has recently advocated the use of the dull curette in puerperal septicæmia. Where the intrauterine douche fails to reduce temperature, he scrapes the cavity of the womb thoroughly and follows it by another douching. Dr. Grandin believes that the endometrium becomes necrotic in these cases and requires more than a washing for its removal. This treatment has been generally well received and several cases have lately given opportunity to test its value. Only a few days ago a physician told me that his patient's temperature remained unaffected by douchings, but the curette brought away a large quantity of a very tenacious gummy-like material, which the injections could not possibly have removed. After this the temperature fell and general improvement and recovery followed.

The Middleton Goldsmith Lecture this year will be delivered by Dr. Reginald H. Fitz, Shattuck Professor of Pathology in the Harvard Medical School. The subject will be: Acute Pancreatitis, with an especial consideration of Pancretaic Hæmorrhage, Hæmorrhagic Pancreatitis and Subperitoneal Fat Necrosis.

New York, Feb. 8th, 1889. A. F.

